# Short-Term Inhalation of Ultrafine Zinc Particles Could Alleviate Cardiac Dysfunctions in Rats of Myocardial Infarction

**DOI:** 10.3389/fbioe.2021.646533

**Published:** 2021-04-14

**Authors:** Li Li, Pei Niu, Xuan Wang, Fangbo Bing, Wenchang Tan, Yunlong Huo

**Affiliations:** ^1^Department of Mechanics and Engineering Science, College of Engineering, Peking University, Beijing, China; ^2^PKU-HKUST Shenzhen-Hong Kong Institution, Shenzhen, China; ^3^Peking University Shenzhen Graduate School, Shenzhen, China; ^4^Shenzhen Bay Laboratory, Shenzhen, China; ^5^Institute of Mechanobiology and Medical Engineering, School of Life Sciences and Biotechnology, Shanghai Jiao Tong University, Shanghai, China

**Keywords:** speckle-tracing echocardiography, strain analysis, ultrafine zinc particle, Womersley analysis, myocardium infraction

## Abstract

It is not clear for inhalation of ultrafine metal particles in air pollution to impair human health. In the study, we aimed to investigate whether short-term (4 weeks) inhalation of ultrafine zinc particles could deteriorate the cardiac and hemodynamic functions in rats of myocardial infarction (MI). MI was induced in Wistar rats through coronary artery ligation surgery and given an inhalation of ultrafine zinc particles for 4 weeks (post-MI 4 weeks, 4 days per week, and 4 h per day). Cardiac strain and strain rate were quantified by the speckle tracking echocardiography. The pressure and flow wave were recorded in the carotid artery and analyzed by using the Womersley model. Myocardial infarction resulted in the LV wall thinning, LV cavity dilation, remarkable decrease of ejection fraction, dp/dt Max, −dp/dt Min, myocardial strain and strain rates, and increased LV end-diastolic pressure, as well as impaired hemodynamic environment. The short-term inhalation of ultrafine zinc particles significantly alleviated cardiac and hemodynamic dysfunctions, which could protect from the MI-induced myocardial and hemodynamic impairments albeit it is unknown for the long-term inhalation.

## Introduction

The adverse effects of ambient air pollution in the pathogenesis of acute and chronic diseases are recognized increasingly. The ultrafine metal particles in air pollution have the possibilities to deteriorate the cardiovascular diseases (Birmili et al., 2006; Kodavanti et al., 2008; Wallenborn et al., 2008). Zinc is one of the main metal elements in air pollution in China (Ming et al., 2017; Du et al., 2019). Zinc is also a ubiquitous trace element. It is one of the most important and indispensable trace elements in the body, and it is involved in the growth and development of microorganisms, plants, and animals (Chasapis et al., 2012). Zinc ions (Zn^2+^) plays an important role in the excitation-contractile coupling of mammalian cardiomyocytes (Tuncay et al., 2011). Hence, it is worthwhile to study the effect of ultrafine zinc particles on cardiovascular diseases.

We have previously shown that the inhalation of ultrafine zinc particles deteriorated local myocardial dysfunctions in the LV and the hemodynamic environment in peripheral arteries in rats of hypertension-induced heart failure with preserved ejection fraction (HFpEF) (Bing et al., 2020). The zinc level increased in the blood and tissues of hypertensive rats after inhalation of zinc particles (Clegg et al., 1987; Leblondel and Allain, 1988; Henrotte et al., 1990). ZIP14 is a plasma membrane transporter that promotes extracellular zinc to enter the cytoplasm and increases the zinc concentration inside the cell (Taylor et al., 2005). Inhaling zinc particles upregulated zinc transporter ZIP14 expression (Huang J. Y. et al., 2018) and induced accumulation of intracellular Zn^2+^ in the myocytes, which resulted in the impaired excitation-contraction coupling of myocytes in hypertension-induced HFpEF (Macdonald, 2000; Murakami and Hirano, 2008). In contrast, a significant fall in serum zinc levels was observed in patients with acute myocardial infarction (MI) that induced heart failure with reduced ejection fraction (HFrEF) (Lewandowicz et al., 1979; Singh et al., 1983). Since there are totally different biological and hemodynamic mechanisms between HFpEF and HFrEF, we hypothesized that short-term inhalation of ultrafine zinc particles could slow down the progression of cardiac and hemodynamic impairments in rats of MI.

The objective of the study is to investigate cardiac and hemodynamic changes in rats of MI after inhaling ultrafine zinc particles. Wistar rats were selected for coronary artery ligation surgery to induce MI as well as inhalation of ultrafine zinc particles for 4 weeks. Physiological and hemodynamic measurements were carried out in the LV and carotid artery for 4 weeks after the ligation surgery. Speckle tracking echocardiography (STE) was used to analyze the ventricular functions (Voigt et al., 2015). The Womersley model was performed for the hemodynamic analysis in the carotid artery. The significance and implications of the study were discussed relevant to ultrafine zinc particles’ protection effect on the MI-induced myocardial and hemodynamic impairments.

## Materials and Methods

### Study Design

**FIGURE 1:**
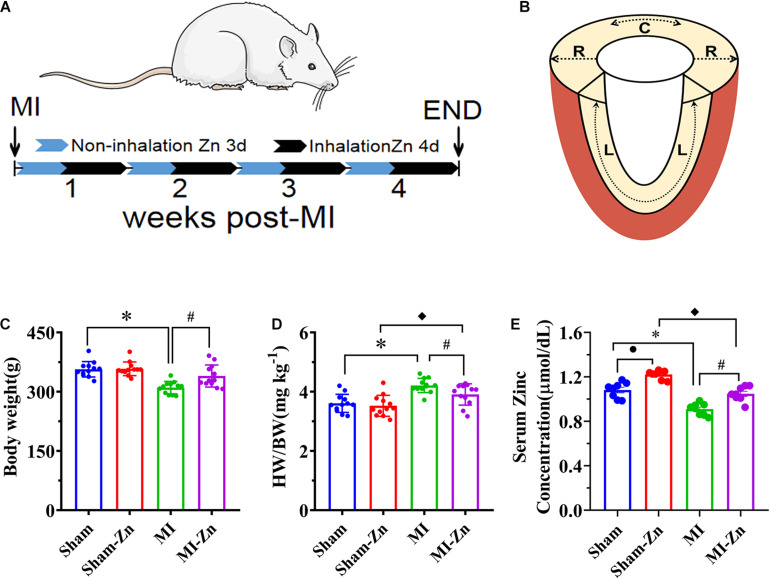
**(A)** Schematic representative of experimental protocol, where Sham-Zn and MI-Zn groups were exposed to ultrafine zinc particle for 4 days per week in 4 weeks after LAD ligation. **(B)** Schematic representative of myocardium strain in radial, longitudinal, and circumference directions. **(C)** Body weight, **(D)** the ratio of HW to BW, and **(E)** serum zinc concentration in Sham (*n* = 12), Sham-Zn (*n* = 12), MI (*n* = 12), and MI-Zn (*n* = 12) groups at postoperative 4 weeks. ^∗^*P* < 0.05, MI vs. Sham. ^•^*P* < 0.05, Sham vs. Sham-Zn. ^◆^*P* < 0.05, MI-Zn vs. Sham-Zn. ^#^*P* < 0.05, MI-Zn vs. MI.

A total of 80 male 8-week-old Wistar rats (Beijing Vital River Laboratory Animal Technology Co., Ltd.), weighing 208 ± 14 g, were used for this study. Rats were housed at standard SPF laboratory and free access to standard rodent chow and water. Randomly, 30 rats were divided into sham (sham group, *n* = 15) and sham with inhalation of zinc particles (sham-Zn group, *n* = 15) and 50 rats were performed with left anterior descending artery (LAD) ligation surgery to induce MI. Six animals died during the surgery. Three days after surgery, half of the surviving MI rats (*n* = 22) were exposed in the environment filled with ultrafine zinc particle (diameter of 50 nm and density of 500 μg/m^3^) (MI-Zn group, *n* = 22) using the same method as a previous study (Bing et al., 2020) while the rest were considered as the MI group (*n* = 22). MI-Zn and Sham-Zn rats inhaled ultrafine zinc particles for 4 h per day and 4 days per week for 4 weeks, as shown in Figure 1A. All experiments were performed in accordance with Chinese National and Peking University ethical guidelines regarding the use of animals in research, consistent with the NIH guidelines (Guide for the care and use of laboratory animals) on protection of animals used for scientific purposes. The experimental protocol was approved by the Animal Care and Use Committee of Peking University, China.

### Myocardial Infarction

Left anterior descending artery ligation was performed in 50 Wistar rats. Briefly, in sterile environment, surgical anesthesia was maintained with ∼2% isoflurane and animals were intubated and ventilated with room air and oxygen using a Harvard ventilator (Inspira) (Niu et al., 2020). After the chest was shaved and sterilized, a left thoracotomy was performed between the third and fourth intercostal spaces. A 7-0 suture line was placed at 1 mm below the left auricle appendage to ligate the LAD artery, which led to pale LV anterior wall and apex region. Alternatively, the suture was placed but removed in sham-operated animals. After the chest was closed, animals were intramuscularly administered a dose of penicillin (400,000 U) and 1 ml dezocine (50 μg/ml) and allowed to recover from the surgery. Animals were given an intramuscular injection of penicillin (400,000 U) and 1 ml dezocine (50 μg/ml) for three consecutive days. All animals were cared at 26°C indoors, and under 12:12 h light/dark artificial cycle conditions for a total of 4 weeks after surgical recovery.

### Echocardiographic Measurements

Echocardiographic measurements of rat hearts, as shown in Figure 1B, were carried out under anesthesia for 4 weeks postoperatively. M-mode measurements of LV, left atrium, and aorta and B-mode measurements of strain and strain rate were recorded in rats, similar to a previous study (Niu et al., 2020). The images were obtained at 21 MHz using a MS-250 transducer operated by a Vevo 2100 Color Doppler Ultrasound Scanner (FUJIFILM VisualSonics Inc.). Based on M-mode tracings, morphometric parameters, e.g., LVID;d, LVID;s, LVFW;s, LVFW;d, IVS;s, and IVS;d, were measured according to the American Society of Echocardiography leading edge rule (Sahn et al., 1978). These parameters were averaged based on five measurements. Moreover, FS (%) and EF (%) were calculated from the measured parameters as: $\frac{\left(L\,V\,I\,D;d-L\,V\,I\,D;s\right)}{L\,V\,I\,D;d}\times 100\%$ and $\frac{\left(L\,V\,I\,D;d^{3}-L\,V\,I\,D;s^{3}\right)}{L\,V\,I\,D;d^{3}}\times 100\%$, respectively, on a Vevo LAB image analysis workstation.

### STE Analysis

Myocardial deformation and movement measurements were carried out by using the Vevo LAB image analysis workstation with advanced STE, which tracks natural acoustic markers (called speckles) across the cardiac cycle and estimates velocity vectors. Strain measurement of myocardial deformation were obtained from B-Mode cine loops acquired from the parasternal long-axis and short-axis views (Niu et al., 2020). Frame rate is 133 Hz, gain is 20∼25 dB, depth is ∼20 mm, width is ∼23 mm, and three cardiac cycles were recorded. Longitudinal and circumferential stains (S = $\frac{\Delta \,L}{L_{0}}$, where *L*_*0*_ and Δ*L* refer to the baseline length at the R-Wave and the absolute change in length, respectively) and strain rate (SR = $\,\frac{S}{\Delta \,t}$ = $\,\frac{\left(\Delta \,L/L_{0}\right)}{\Delta \,t}$ = $\frac{\left(\Delta \,L/\Delta \,t\right)}{L_{0}}$= $\frac{\Delta \,V}{L_{0}}$, where Δ*V* is the velocity gradient in the segment) were determined by the software across a selected period of cardiac cycles.

### Hemodynamic Measurements

The left carotid artery (LCA) was dissected in a sterile environment under anesthesia after the echocardiographic measurements. A perivascular flow probe (Transonic Systems Inc.; relative error of ±2% at full scale) was used to measure volumetric flow rate of LCA. Moreover, a 1.4F micromanometer-tipped catheter (Millar Instruments) was inserted through the right carotid artery into the LV to record pressure waves over 30 cardiac cycles, which was repeated three times. The zero-pressure baseline of the catheter was calibrated in the 37°C saline solution. The catheter and perivascular flow probe were monitored with a BIOPAC MP150. Heart rate, LV systolic pressure (LVSP), LV end-diastolic pressure (LVEDP), and rate of maximum positive and negative left ventricular pressure development (${\frac{\text{dp}}{\text{dt}}}_{\text{max}}$ and ${\frac{\text{dp}}{\text{dt}}}_{\text{min}}$) were determined from the measured pressure waves.

### Serum Zinc Detection

After hemodynamic measurements, blood samples were taken from the tail vein and centrifuged at 3000 RPM for 15 min. The serum was extracted and stored at −20°C. The Blood Zinc Concentration Detection Kit (Solarbio BC2815, Beijing Solarbio Science & Technology Co., Ltd.) was used to detect zinc concentration in the serum, which was measured by the microplate reader (Multiskan^TM^ FC, Thermo Fisher Scientific, United States) with a wavelength of 620 nm.

### Histological Evaluation

All animals were terminated for the histological analysis at postoperative 4 weeks by intraperitoneal injection of 1% pentobarbital sodium at dose of 150 mg/kg (Niu et al., 2020). After hearts were harvested, plugs of myocardial tissues were removed from different positions of the LV. These plugs were fixed in 4% paraformaldehyde (PFA)/PBS solution overnight at room temperature and then processed for paraffin sectioning. Masson’s trichrome staining was carried out for determination of myocardial fibrosis according to standard procedures (Puente et al., 2014; Deng et al., 2016) while haematoxylin-eosin (HE) staining was performed to observe the arrangement and morphology of cardiomyocytes (Wu et al., 2017).

### Womersley Analysis

Similar to a previous study (Bing et al., 2020), the equation for the pulsatile flow velocity profile across the lumen, *u*(*r*,*t*), is given as:

(1) $$u\,\left(r,t\right)=\text{REAL}\,\left(\frac{2\,Q\,\left(0\right)\,\left(R^{2}-r^{2}\right)}{\pi \,R^{4}}+\sum_{\omega =1}^{\infty }\frac{\frac{Q\,\left(\omega \right)}{\pi \,R^{4}}\cdot \left(1-\frac{J_{0}\,\left(\Lambda \,r/R\right)}{J_{0}\,\left(\Lambda \right)}\right)}{1-\frac{2\,J_{1}\,\left(\Lambda \right)}{\Lambda \,J_{0}\,\left(\Lambda \right)}}\,e^{i\,\omega \,t}\right)$$

where *r* is the radial coordinate, *R* is the radius of artery, Λ^2^ = *i*^3^α^2^, $\alpha =\text{R}\,\sqrt{\frac{\omega \,\rho }{\mu }}$, *q*_*measured*_(t) = Q(ω)*e*^*i*ω*t*^, ω is the angular frequency after Fourier transformation, *J*_0_is a Bessel function of zero order and first kind, and *J*_*1*_ is a Bessel function of first order and first kind. Accordingly, wall shear stress (WSS), τ(R,t), and oscillatory shear index (OSI) for pulsatile blood flow can be written as:

(2) $$\tau \,\left(\text{R},\text{t}\right)=\text{REAL}\,\left(\frac{4\,\mu }{\pi \,R^{3}}\,Q\,\left(0\right)-\sum_{\omega =1}^{\infty }\frac{\frac{\mu \,Q\,\left(\omega \right)}{\pi \,R^{3}}\cdot \frac{\Lambda \,J_{1}\,\left(\Lambda \right)}{J_{0}\,\left(\Lambda \right)}}{1-\frac{2\,J_{1}\,\left(\Lambda \right)}{\Lambda \,J_{0}\,\left(\Lambda \right)}}\right)$$

(3) $$\mathrm{OSI}=\frac{1}{2}\,\left(1-\frac{\left|\frac{1}{T}\,\int_{0}^{T}\tau \,\left(\text{R},\text{t}\right)\right|}{\frac{1}{T}\,\int_{0}^{T}\left|\tau \,\left(\text{R},\text{t}\right)\right|}\right)$$

The viscosity (μ) and density (ρ) were assumed to be 4.0 cp and 1.06 g/cm^3^, respectively. Moreover, relative residence time (RRT) reflects the residence time of flow particles near the wall and is recommended as a single metric of low oscillating shear stress, which is expressed as follows:

(4) $$\mathrm{RRT}=\frac{1}{\left(1-2\cdot O\,S\,I\right)\cdot T\,A\,W\,S\,S}$$

### Statistical Analysis

The experimental measurements were repeated three times and averaged per animal. All parameters were represented as mean ± S.E.M. by averaging over all animals in each group. A two-way ANOVA (SigmaStat 3.5) was used to detect the statistical difference of morphometric and hemodynamic parameters between sham and MI groups and between inhalation of zinc particle and no inhalation groups, where *P* < 0.05 was indicative of a significant difference between the two populations.

## Results

Figures 1C,D show the body weight and ratio of HW to BW in Sham, Sham-Zn, MI, and MI-Zn groups at postoperative 4 weeks, which shows no significant difference between Sham (BW: 357 ± 20 g, HW/BW: 3.59 ± 0.32) and Sham-Zn group (BW: 358 ± 17 g, HW/BW: 3.54 ± 0.37). MI-Zn rats have higher BW (340 ± 28 g) and lower HW/BW (3.89 ± 0.37) than the MI group (BW: 310 ± 16 g, HW/BW: 4.21 ± 0.25). The MI group has a significant decrease of BW and an increase of HW/BW than the Sham group and the MI-Zn group has higher HW/BW than the Sham-Zn group. Accordingly, Figure 1E shows the serum zinc concentration in the four groups. Myocardial infarction reduced the zinc concentration significantly while inhaling ultrafine zinc particle increased it.

Table 1 lists morphometric and hemodynamic parameters in the heart of the four groups. There is no statistical difference between Sham and Sham-Zn groups except for LV wall thickness. In comparison with Sham and Sham-Zn groups, LVID;s, LVID;d, ESV, EDV, and LVEDP are significantly higher and LV free wall thickness and systolic and diastolic blood pressures are lower in MI and MI-Zn groups. On the other hand, the MI-Zn group has significantly higher systolic blood pressure and lower LV end-diastolic pressure than the MI group.

**TABLE 1:** Morphometric and hemodynamic parameter.

	**Sham**	**Sham-Zn**	**MI**	**MI-Zn**
SV (μl)	174 ± 70.0	187 ± 34.1	143 ± 20.4	170 ± 55.3
CO (ml/min)	65.0 ± 21.4	73.8 ± 17.4	48.9 ± 7.12	60.2 ± 29.2
LVID;s (mm)	3.42 ± 0.53	3.28 ± 0.41	9.17 ± 0.47*	8.84 ± 0.72^#◆^
LVID;d (mm)	6.29 ± 0.43	6.66 ± 0.54	10.4 ± 0.82*	9.98 ± 0.73^◆^
ESV (μl)	50.0 ± 17.7	43.5 ± 13.5	468 ± 53.0*	396 ± 74.7^#◆^
EDV (μl)	202 ± 30.7	230 ± 43.1	618 ± 107*	566 ± 91.3^◆^
LVAW;s (mm)	2.56 ± 0.30	2.97 ± 0.12^•^	1.07 ± 0.11*	1.15 ± 0.42^◆^
LVAW;d (mm)	1.67 ± 0.14	1.92 ± 0.05^•^	1.03 ± 0.11*	1.19 ± 0.39^◆^
LVPW;s (mm)	2.50 ± 0.24	3.01 ± 0.21^•^	2.56 ± 0.37	2.60 ± 0.57
LVPW;d (mm)	1.67 ± 0.18	2.02 ± 0.18^•^	1.91 ± 0.25*	1.95 ± 0.26
LVSP (mmHg)	124 ± 14.7	127 ± 11.4	101 ± 23.63*	110 ± 11.9^◆^
LVEDP (mmHg)	1.29 ± 0.88	3.62 ± 4.01	17.8 ± 9.68*	10.1 ± 4.78^#◆^
SBP (mmHg)	134 ± 15.2	140 ± 5.32	106 ± 6.34*	114 ± 4.99^#◆^
DBP (mmHg)	100 ± 16.2	116 ± 6.98	86 ± 11.0*	89.3 ± 8.83

^•^*P* < 0.05, Sham vs. Sham-Zn, *P < 0.05, MI vs. Sham, ^◆^*P* < 0.05, Sham-Zn vs. MI-Zn, and ^#^*P* < 0.05, MI-Zn vs. MI.

**FIGURE 2:**
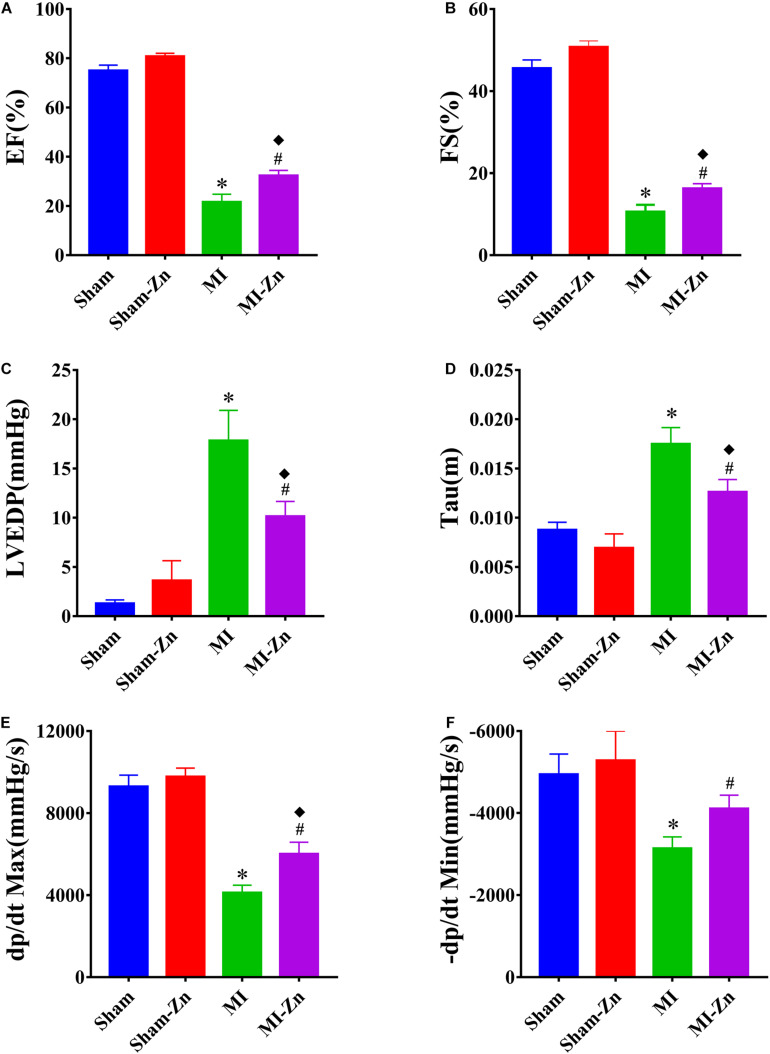
**(A)** EF (%), **(B)** FS (%), **(C)** LVEDP (mmHg), **(D)** Tau, **(E)** dp/dt Max, and **(F)** –dp/dt Min (mmHg/s) in Sham (*n* = 12), Sham-Zn (*n* = 12), MI (*n* = 12), and MI-Zn (*n* = 12) groups at postoperative 4 weeks. ^∗^*P* < 0.05, MI vs. Sham. ^◆^*P* < 0.05, MI-Zn vs. Sham-Zn. ^#^*P* < 0.05, MI-Zn vs. MI.

Figure 2 shows EF, FS, LVEDP, Tau, dp/dt Max, and −dp/dt Min in four groups at postoperative 4 weeks, which have no statistical difference between Sham and Sham-Zn groups, but significant difference between Sham and MI groups and between Sham-Zn and MI-Zn groups. Moreover, the MI-Zn group has higher values of EF, FS, dp/dt Max, and −dp/dt Min and lower values of LVEDP and Tau as compared with the MI group.

**FIGURE 3:**
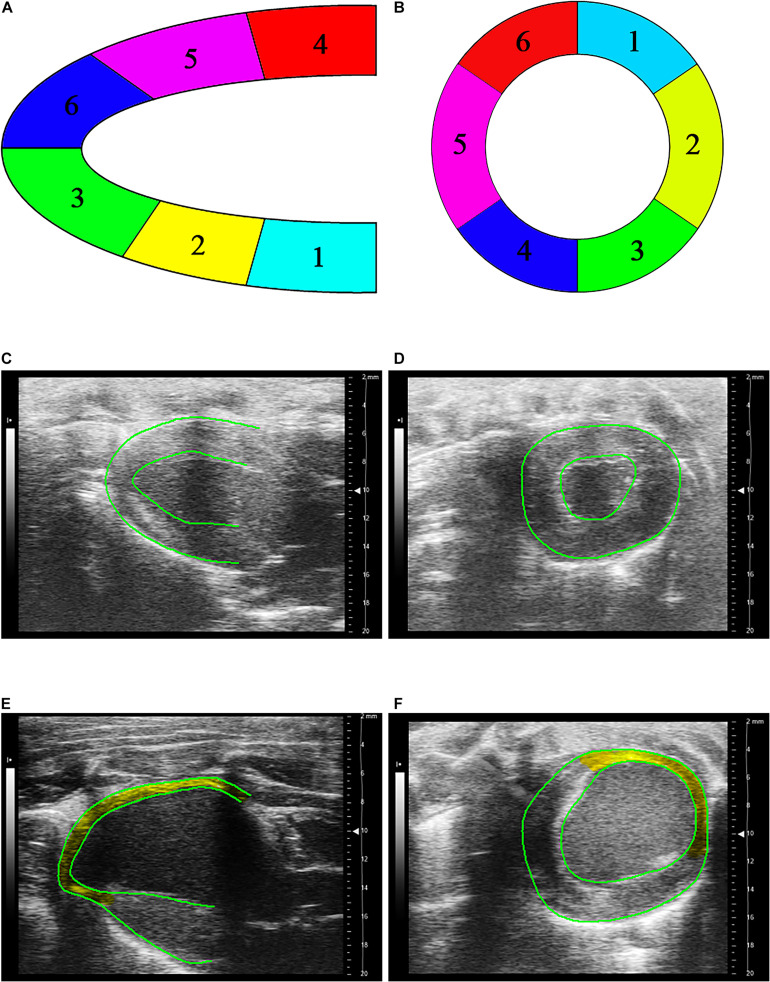
**(A)** Schematic representative of deformation analysis in six segments along the long-axis echocardiographic view, where segments 1–6 refer to LV basal posterior wall, middle posterior wall, posterior apex, basal anterior wall, middle anterior wall, and anterior apex, respectively, **(B)** schematic representative of deformation analysis in six segments along the short-axis echocardiographic view, where segments 1–3 refer to free wall and segments 4–6 refer to interventricular septum, **(C)** long-axis, and **(D)** short-axis echocardiographic views in a representative Sham, **(E)** long-axis, and **(F)** short-axis echocardiographic views in a representative of MI, where yellow region marks the infarction area.

Figure 3 shows schematic representative of deformation analysis and long-axis and short-axis echocardiographic views in four groups. In comparison with Sham and Sham-Zn groups, peak values of longitudinal, circumferential, and radial strain and strain rates in both infarction and normal regions are significantly reduced in MI and MI-Zn groups, as shown in Table 2. There is no statistical difference of strain and strain rates between Sham and Sham-Zn groups. Peak values of longitudinal, circumferential and radial strain and strain rates in infarction and normal regions of MI and MI-Zn groups are significantly lower than those of Sham and Sham-Zn groups. While peak values of three directions’ strain and strain rates in infarction and normal regions of the MI-Zn group are higher than those in the MI group.

**TABLE 2:** Myocardial strain and strain rate derived by echocardiography.

	**Normal zone**				**Infraction zone**			
	**Sham**	**Sham-Zn**	**MI**	**MI-Zn**	**Sham**	**Sham-Zn**	**MI**	**MI-Zn**
L-Strain	−18.16 ± 2.57	−19.75 ± 4.27	−7.39 ± 4.29*	−11.19 ± 3.75^◆^**^#^**	−15.36 ± 2.99	−15.12 ± 3.56	−4.55 ± 1.95*	−5.27 ± 2.27^◆^
L-Strain rate	−4.15 ± 0.72	−4.85 ± 1.45	−2.52 ± 0.86*	−3.15 ± 0.51^◆^**^#^**	−3.25 ± 0.67	−3.77 ± 0.96	−1.57 ± 0.42*	−1.86 ± 0.52^◆^
C-Strain	−22.06 ± 3.35	−22.12 ± 6.86	−10.09 ± 5.00*	−14.90 ± 3.99^◆^**^#^**	−19.57 ± 5.03	−18.80 ± 6.42	−2.83 ± 2.35*	−7.97 ± 2.50^◆^**^#^**
C-Strain rate	−4.86 ± 0.84	−5.91 ± 2.16	−2.64 ± 0.84*	−3.48 ± 1.03^◆^**^#^**	−4.47 ± 1.45	−4.81 ± 1.37	−1.21 ± 0.43*	−1.60 ± 0.34^◆^**^#^**
R-Strain	41.51 ± 6.29	39.21 ± 16.35	14.21 ± 7.23*	27.95 ± 3.65^◆^**^#^**	32.95 ± 9.42	39.46 ± 9.93	7.31 ± 5.06*	20.55 ± 5.26^◆^**^#^**
R-Strain rate	6.02 ± 0.82	6.35 ± 1.16	3.93 ± 0.76*	5.20 ± 0.86^◆^**^#^**	5.48 ± 1.17	6.78 ± 1.59	3.13 ± 1.11*	4.45 ± 1.24^◆^**^#^**

L, Longitudinal; C, Circumferential; R, Radial. *P < 0.05, MI vs. Sham; ^◆^*P* < 0.05, Sham-Zn vs. MI-Zn; **^#^***P* < 0.05, MI-Zn vs. MI.

**FIGURE 4:**
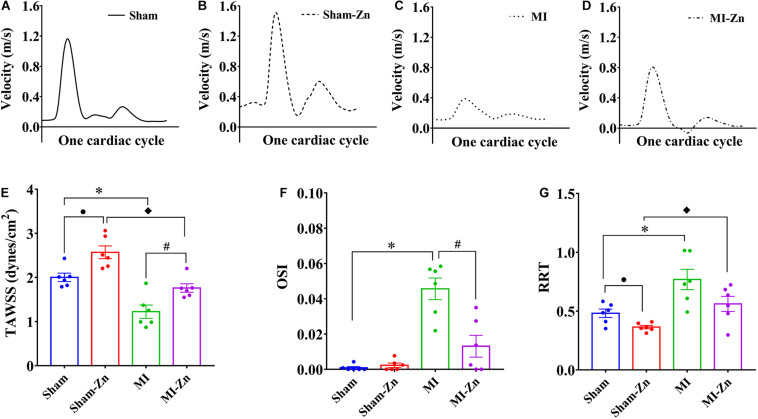
**(A–D)** Transient distribution of representative flow velocity in the carotid artery of **(A)** Sham, **(B)** Sham-Zn, **(C)** MI, and **(D)** MI-Zn groups at postoperative 4 weeks; and **(E)** TAWSS (dynes/cm^2^), **(F)** OSI, and **(G)** RRT in the carotid artery of Sham (*n* = 6), Sham-Zn (*n* = 6), MI (*n* = 6), and MI-Zn (*n* = 6) groups at postoperative 4 weeks. ^•^*P* < 0.05, Sham vs. Sham-Zn. ^∗^*P* < 0.05, MI vs. Sham. ^◆^*P* < 0.05, MI-Zn vs. Sham-Zn. ^#^*P* < 0.05, MI-Zn vs. MI.

Figures 4A–D show transient distribution of representative flow velocity in the carotid artery of four groups at postoperative 4 weeks. Accordingly, Figures 4E–G show mean values of TAWSS, OSI, and RRT. Sham-Zn and MI-Zn groups have higher TAWSS and lower RRT than the Sham and MI groups, respectively. The MI group has the highest OSI.

**FIGURE 5:**
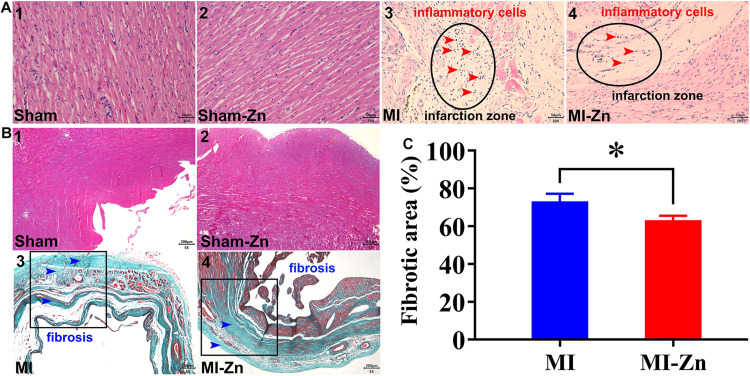
**(A,B)** Schematic representative of HE **(A)** and Masson trichromatic **(B)** staining. The results of HE staining were observed under 20× objective lens (scale label: 50 μm), and Masson trichromatic staining was observed under 5× objective lens (scale label: 200 μm). **(C)** Quantitative comparison of myocardium fibrosis between MI group and MI-Zn group. ^∗^*P* < 0.05, MI vs. MI-Zn.

Figures 5A,B show HE and Masson trichromatic staining in the myocardium of four groups at postoperative 4 weeks. Accordingly, Figure 5C shows quantitative comparison of myocardium fibrosis between MI group and MI-Zn group. Myocardial infarction leads to a significant increase of myocardium fibrosis, which is inhibited by short-term inhalation of ultrafine zinc particles.

## Discussion

This study investigated the effects of inhaling ultrafine zinc particles on cardiac function and peripheral cardiovascular hemodynamics in normal and MI animals experimentally and theoretically. The findings revealed that short-term inhalation of ultrafine zinc particles could inhibit the progression of the MI-induced heart failure.

### LV Dysfunctions and Remodeling

Myocardial infarction resulted in cardiac impairments, such as LV wall thinning, LV cavity dilation, remarkable decrease of EF, FS, dp/dt Max, and −dp/dt Min, and increased LVEDP and Tau as well as decreased systolic and diastolic blood pressures for 4 weeks after LAD ligation. The short-term inhalation of ultrafine zinc particles slowed down cardiac impairments caused by the myocardial infarction. The role of zinc as a modulator of cardiac changes has been recognized, which could affect homeostasis, oxidative stress, immune function, apoptosis, and ageing (Chasapis et al., 2012; Andrea et al., 2018). Zinc, a vital antioxidative element, can protect myocyte membrane against unsaturated lipids and inflammatory cytokines (Hennig et al., 1996) and thus alleviate the infarction-induced heart failure (Shokrzadeh et al., 2009) albeit there was much debate about the role of zinc concentrations in different types of cardiomyopathy (Marin and Rodriguez-Martinez, 1995; Kosar et al., 2006). A meta-analysis indicated a significant association between zinc deficiency and MI (Liu et al., 2015). The short-term inhalation of ultrafine zinc particles alleviated myocardial dysfunctions and slowed down conversion of MI to heart failure in MI rats, which agreed with previous studies that EF increased with Zn content (Oster et al., 1993) and Zn supplementation to a cardioplegic solution reversed the loss of systolic function (Powell et al., 1995).

Myocardial fibrosis makes a critical contribution to the LV remodeling in MI-induced HFrEF. Ultrafine zinc particles entered the systemic circulation through the respiratory system and suppressed tissue collagen deposition by inhibiting proline hydroxylase activity (Mezey et al., 1976; Mann et al., 1979; Camps et al., 1992), and regulated metabolism of fiber collagen (Wang et al., 2005). Wang et al. (2005) have shown that myocardial fibrosis was related to metallothionein (MT) regulation of zinc homeostasis and zinc supplementation prevented the fibrotic process in the MT-KO mice. Inhaling ultrafine zinc particles reduced the degree of myocardial fibrosis and hence slowed down conversion of MI to heart failure in MI rats.

The MI group had a significant decrease of systolic and diastolic blood pressures. The short-term inhalation of ultrafine zinc particles stimulated peripheral arteries and arterioles to elevate systolic and diastolic blood pressures in the MI-Zn group, which can be another factor for Zn protection against the MI-induced heart failure. On the other hand, we have shown a significant increase of systolic and diastolic blood pressures in rats of HFpEF (Bing et al., 2020). The short-term inhalation of ultrafine zinc particles further increased the systolic and diastolic blood pressures and hence deteriorated cardiac and hemodynamic environment in rats with HFpEF.

### Cardiac Strain Analysis

Myocardial strain and strain rate characterize the extent of cardiac deformation (Niu et al., 2020). In the STE analysis, the longitudinal strain refers to the shortening of myocardium fibers from the base to the apex, the circumferential strain represents the circumferential shortening observed in the short-axis view (Saito et al., 2009), and the radial strain shows the myocardial shortening moving from the LV center to the periphery (Heimdal, 1998). Accordingly, the strain rates refer to the relaxation of myocardium fibers (Niu et al., 2020). The MI-induced decrease of longitudinal, circumferential and radial strains and strain rates denoted the significant impairment of systolic and diastolic functions, respectively. The short-term inhalation of ultrafine zinc particles slowed down the progression of systolic and diastolic dysfunctions. In mammalian myocytes, it is known that Zn^2+^ is of importance to regulate the excitation-contraction coupling (Turan et al., 1997; Tuncay et al., 2011). Woodier and co-workers showed that cytosolic Zn^2+^ acted as a high affinity activator of RyR2 and modulated the frequency and amplitude of Ca^2+^ waves in myocytes in a concentration-dependent manner (Woodier et al., 2015). Defective Zn^2+^ handling enhanced the impaired contractility in myocytes (Little et al., 2010). Inhaling ultrafine zinc particles significantly increased the serum zinc concentration. Zinc transporter Zip14 can transfer the elevated extracellular zinc into cardiomyocytes (Taylor et al., 2005; Kim et al., 2017; Yusuf et al., 2018) to alleviate systolic and diastolic dysfunctions in MI rats.

### Hemodynamics in Peripheral Arteries

We investigated the hemodynamic changes in the carotid artery of the four groups. The Womersley analysis showed ∼39% reduction of TAWSS in the MI group as compared with the shams. TAWSS in the Sham-Zn group was ∼28% higher than that in the Sham group while the value in the MI-Zn group was ∼44% higher than that in the MI group. This was caused by the increased CO and SV after short-term inhalation of ultrafine zinc particles. Moreover, OSI is high in the MI group despite negligible values in other groups. RRT in the MI group was ∼60% higher than the shams while the value in the MI-Zn group was ∼27% lower than that in MI group. The short-term inhalation of ultrafine zinc particles alleviated the hemodynamic environment in the carotid artery, such as the increased TAWSS, the decreased OSI, and the reduced RRT. These abnormal parameters are known to result in endothelial dysfunction, monocyte deposition, SMC proliferation, microemboli formation, and so on (Huang et al., 2016; Han et al., 2018; Huang X. et al., 2018; Fan et al., 2019). It was reported that MI-induced zinc deficiency aggravates pro-inflammatory and impairs anti-inflammatory responses in vascular endothelial cells though activation of NF-κB and inhibition of PPAR pathways (Connell et al., 1997; Li and Karin, 1999; Chung et al., 2000; Delerive et al., 2000; Hihi et al., 2002; Griendling and FitzGerald, 2003). Zinc supplementation could function as the anti-inflammatory (Jarosz et al., 2017), preventing endothelial cell dysfunction, and subsequent cardiovascular diseases (Shen, 2008). Hence, the short-term inhalation of ultrafine zinc particles may protect peripheral arteries from the hemodynamic impairments, which still required more investigations.

### Critique of the Study

We only measured the serum zinc levels, but not the concentration in myocardial tissues. The proportion of serum zinc entering cardiomyocyte via zinc transporter Zip14 is required to be investigated in the following studies. A further limitation is the lack of inclusion of a control particle, for example, an inert particle of the same size which is identified harmless to body. This is essential to demonstrate that the effects are down to zinc *per se*, rather than simply particulate matter. The present study only considered the effects of short-term inhalation on cardiac function and peripheral cardiovascular hemodynamics. The long-term inhalation of ultrafine zinc particles should be included in the following studies. Moreover, histological analysis and cellular and molecular mechanisms are still required for investigations.

## Conclusion

Myocardial infarction induced cardiac and hemodynamic impairments. The short-term inhalation of ultrafine zinc particles increased EF, FS, cardiac strain, and strain rate as well as decreased LVEDP, which slowed down myocardial dysfunctions in rats of MI. Moreover, the short-term inhalation of ultrafine zinc particles increased TAWSS and decreased OSI and RRT and hence protected peripheral arteries from the hemodynamic impairments.

## Data Availability Statement

The original contributions presented in the study are included in the article/supplementary material, further inquiries can be directed to the corresponding authors.

## Ethics Statement

The animal study was reviewed and approved by the Animal Care and Use Committee of Peking University.

## Author Contributions

LL and PN participated in the design of the study and carried out the animal lab work. LL carried out data statistics analysis and drafted the manuscript. XW and FB carried out the Womersley analysis. WT and YH critically revised the manuscript and conceived and designed the study. All authors contributed to the article and approved the submitted version.

## Conflict of Interest

The authors declare that the research was conducted in the absence of any commercial or financial relationships that could be construed as a potential conflict of interest.

## Abbreviations

HFpEF: Heart failure with preserved ejection fraction
HFrEF: Heart failure with reduced ejection fraction
STE: Speckle-tracing Echocardiography
BW: Body weight
HW: Heart weight
LV: Left ventricle
LAD: Left anterior descending artery
EF (%): Ejection fraction
FS (%): Fractional shortening
SV: Stroke volume
CO: Cardiac output
LVID;s: LV internal diameter in systole
LVID;d: LV internal diameter in diastole
ESV: End- systolic Volume of LV
EDV: End-diastolic Volume of LV
LVAW;s: LV Anterior wall in systole
LVAW;d: LV Anterior wall in diastole
LVPW;s: LV Posterior wall in systole
LVPW;d: LV Posterior wall in diastole
LVSP: LV systolic pressure
LVEDP: LV end-diastolic pressure
LCA: Left Carotid artery
SBP: Systolic blood pressure
DBP: Diastolic blood pressure
TAWSS: Time-average wall shear stress
OSI: Oscillating shear index
RRT: Relative residence time
WSS: Wall shear stress.
